# Rotational panoramic radiographs-unusual triple images

**DOI:** 10.4317/jced.51990

**Published:** 2015-02-01

**Authors:** Shakeel-Ahmed Valai-Kasim, Nathamuni-Rengarajan Krishnaswamy, Biju Tom, Rooban Thavarajah

**Affiliations:** 1 M.D.S., Professor. Department of Orthodontics and Dentofacial Orthopedics, Ragas Dental College and Hospital, Uthandi, Chennai; 2 M.D.S., Morth. RCS[Ed], Dip.NB(ortho), Professor and Head. Department of Orthodontics and Dentofacial Orthopedics, Ragas Dental College and Hospital, Uthandi, Chennai; 3 M.D.S., Assistant professor. Department of Orthodontics and Dentofacial, Orthopedics, Ragas Dental College and Hospital, Uthandi, Chennai; 4 M.D.S., Consultant Oral Pathologist

## Abstract

Currently clinicians advice rotational panoramic radiography (RPR) for preliminary investigation. Despite few inherent limitations, rotational panoramic radiography still remains the diagnostic tool of choice. Abnormal structures such as a supernumerary tooth or a device falling within the certain central regions in conventional RPR images may mislead the clinicians towards an inaccurate diagnosis by producing multiple ghost images. Such cases must be treated with circumspect, and apart from RPR, additional imaging modalities need be employed to provide a judicious interpretation of the clinical situation. Thus this manuscript, we present a case where a paramedian supernumerary tooth which exhibited double ghost images on a conventional RPR. This prompted us to elicit the use of a CBCT and 3 dimensional images to determine the true nature of the problem. We outline the working of the diamond principle behind a conventional RPR which cause the appearance of multiple ghost images. The discerning clinician must be cognizant of the possible positional and analytical errors which may be prevalent in a conventional RPR when viewing structures lying in the palatal region, specifically in the midline while making diagnosis.

** Key words:**CBCT, double image, midline supernumerary, OPG.

## Introduction

Rotational panoramic radiography (RPR) is a commonly employed screening tool in dental and orthodontic practice to provide diagnostic information about the teeth; their axial inclinations and its surrounding structures and attachments (1). The common advantages of RPR are increased overall coverage of dental arches, reduced radiation dose compared to other conventional, sectional 2-dimensional radiographic modalities, minimal infection control procedures and the simplicity and rapidity of the procedure. Apart from central area being magnified by 20 to 30% and few other eccentricities, RPR is a better imaging tool, compared to other standard radiographic position/acquiring techniques. RPR is reported have a central image distortion rate of about 20% compared with the patient’s true anatomy (2).

In a large scale study, it was demonstrated that only 0.8% of all the RPR images reviewed were excellent, 66.2% were diagnostically acceptable, and 33% were unacceptable with the most common faults being positioning errors, low density, and contrast (3).

When a PRP study reveals an additional risk for the patient, 3D diagnostic records are often recommended. The aim of this manuscript is to present a rare clinical situation where RPR misled the diagnosis and 3D study led us to accurate diagnosis.

## Case Report

**Figure 1:**
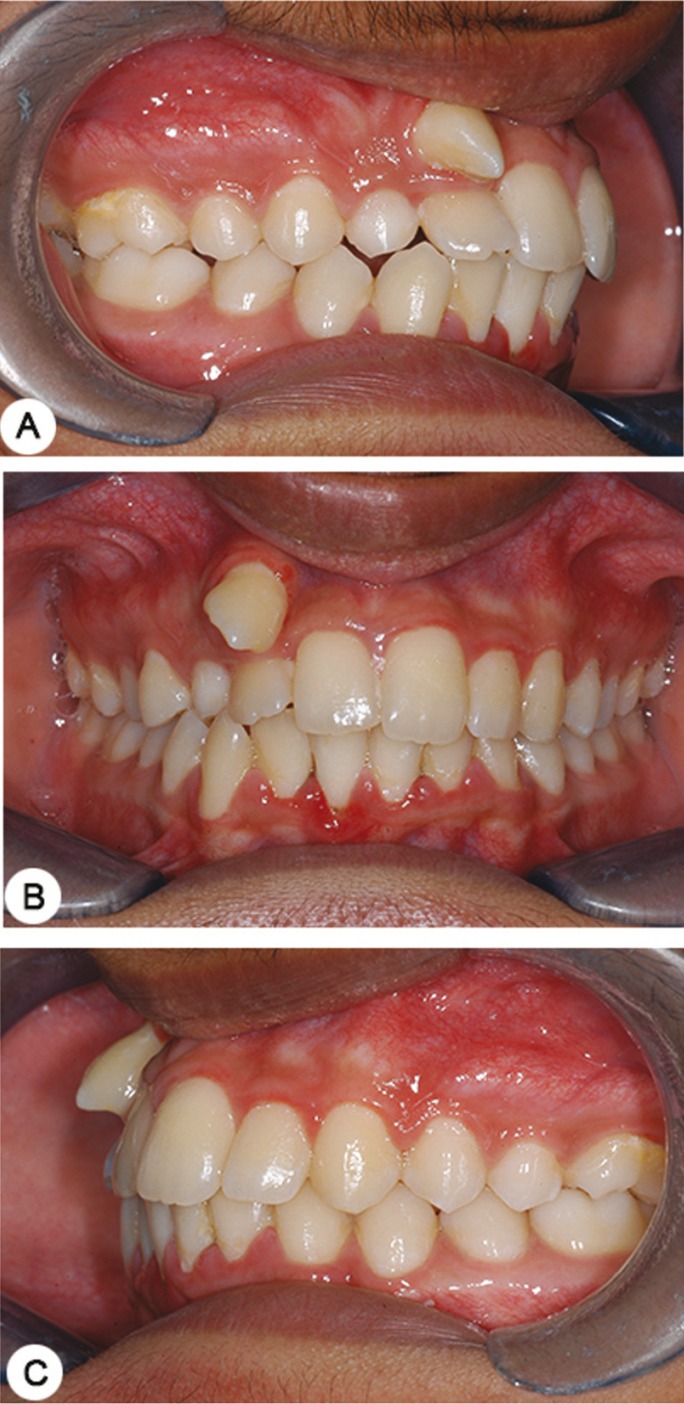
Clinical photographs showing class I malocclusion, retained maxillary right primary canine with transposed maxillary right canine.

**Figure 2:**
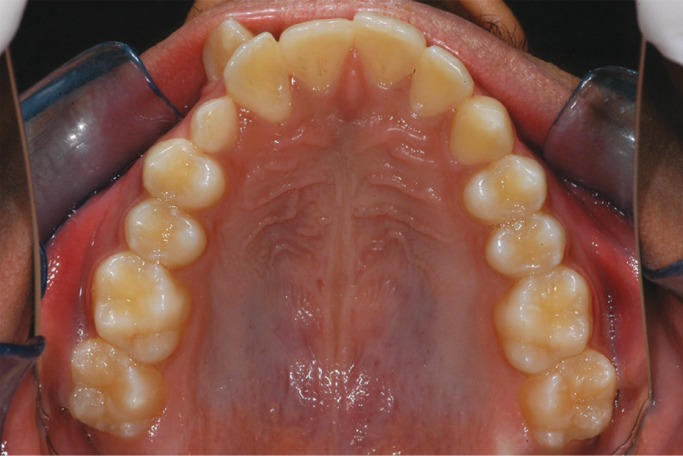
Maxillary occlusal photograph showing retained 53 and trans-positioned 13.

**Figure 3:**
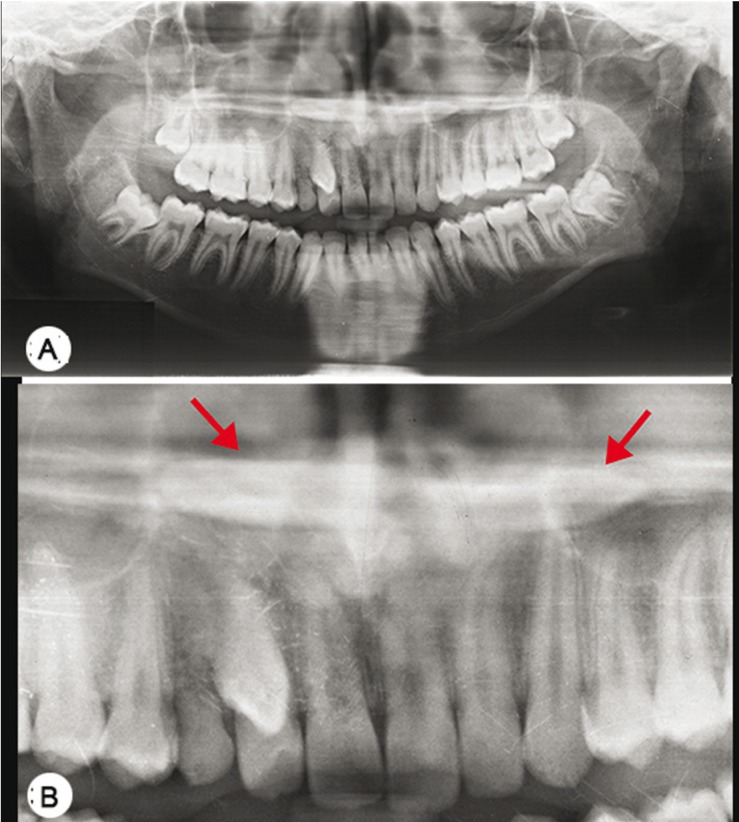
A) Panoramic radiograph showing retained 53 and transposed 13; B) Panoramic radiograph showing shadows of two supernumerary teeth seen above the apices of the anterior sextant.

A 17 year old male patient visited with the complaint of malpositioned upper front teeth and wanted to get them aligned. On clinical examination, he presented with an Angle’s Class I malocclusion (Fig. 1) with a retained 53 and a transpositioned 13 in relation to the same sextant (Fig. 2). A panoramic radiograph shown in figure 3 (Planmeca OY, 00880, Helsinki, Finland, continuous mode) confirmed the presence of a retained 53. Although on clinical examination, the transposed 13 appeared to be mesially directed, the RPR indicated that the root of 13 was distally directed. Further, there were also two supernumerary teeth seen on the right and left palatal region horizontally positioned with the crowns facing laterally in addition to faint multiple images of the palate (Fig. 3).

Due to the conflicting findings from clinical examination and RPR interpretation, further evaluation was performed. We confirmed the transpositioned 13 and its relation with the adjacent teeth and the 2 other supernumerary structures seen over the palatal region with the help of a Cone Beam Computed Tomography (CBCT Kodak C9500 Carestream, 2010) using the standard auto-generated RPR.

As the clinical information, RPR and standard auto generated CBCT acquired RPR view did not coincide, further explorative studies were performed. For this the axial, coronal and sagittal views as well as combined 3D reconstruction of CBCT were extensively studied. From this we identified that there was only a single supernumerary teeth. To identify how a triplication of a single tooth occurred in the auto mode, we altered the focal trough in RPR of CBCT as it identifies the error in using standard auto generated RPR view in CBCT.

**Figure 4:**
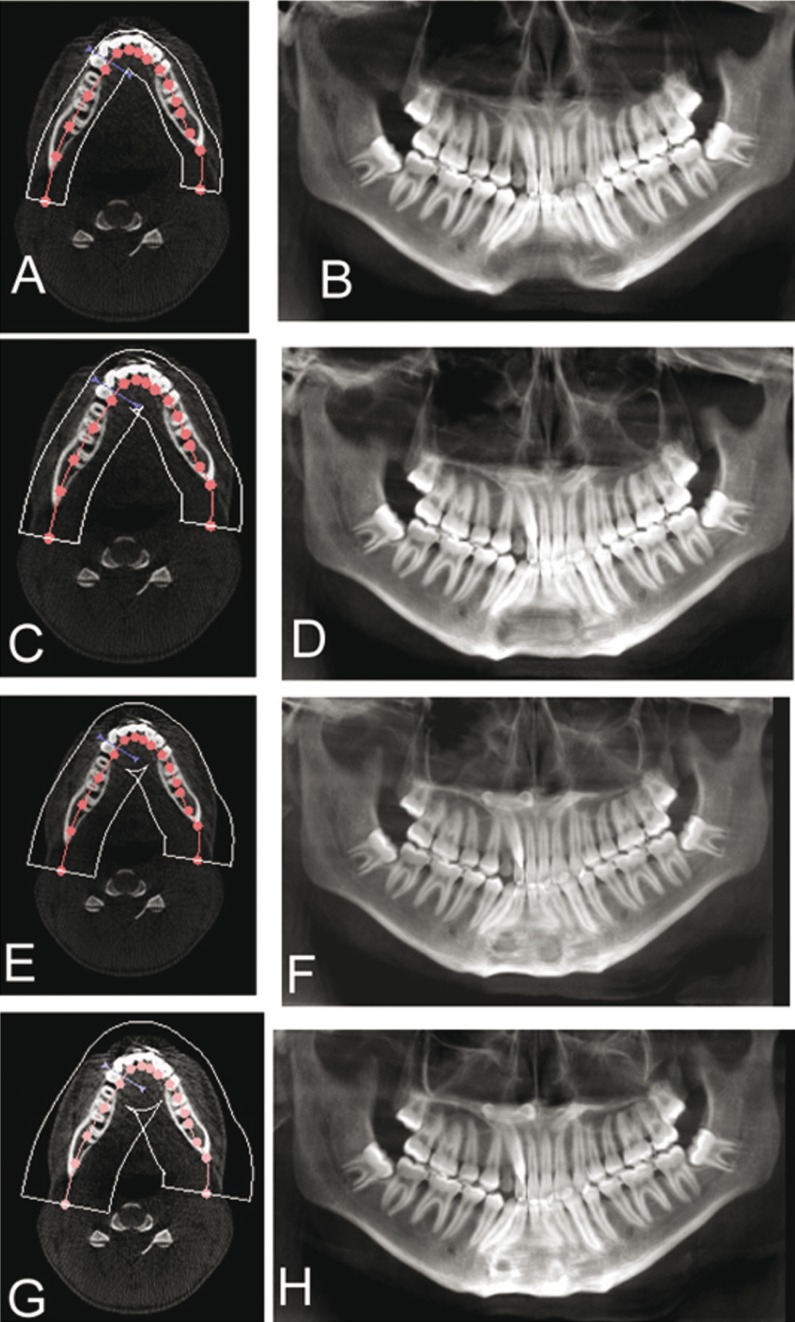
RPR re-constructed from the CBCT with varying trough sizes. Width of the trough: A – 20.1 mm, B (OPG from A); C – 29.7 mm, D (OPG from AC; E – 39.1 mm, F (OPG from E); G – 50.1 mm, H (OPG from H).

**Figure 5:**
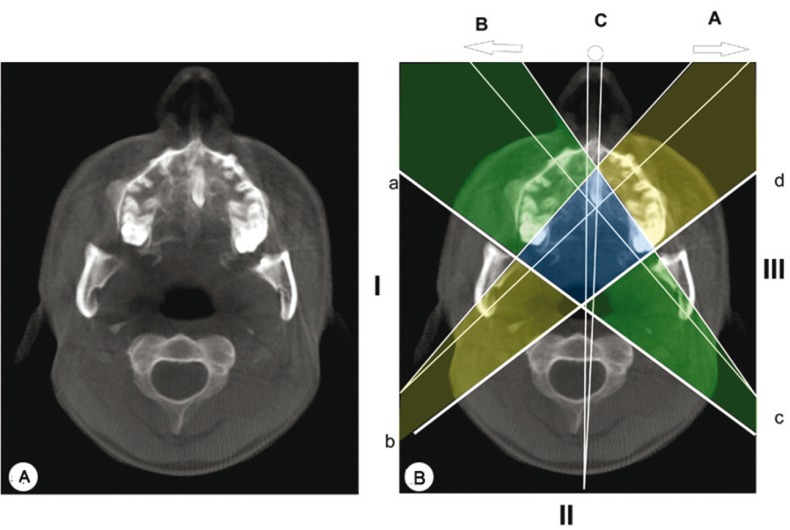
A) A primary reconstruction from the CBCT showing single midline supernumerary in the paramedian region parallel to the midpalatal suture; B) Path of x-rays in a convention RPR, origin to ARC (yellow), the diamond shaped region (blue), ARC to terminal position (green).

Different focal trough sizes were used to form the RPR view from CBCT data. The complete RPR re-constructed (Fig. 4) with a large trough showed two supernumerary structures superimposed over the palatal region while that with a smaller trough exhibited no such image (Fig. 4). However, a primary reconstruction (Fig. 5) evaluation of the same CBCT, revealed only a single supernumerary tooth that was found in the paramedian region parallel to the midpalatal suture, horizontally positioned in the anteroposterior direction with the crown directed posteriorly and root anteriorly. The patient is currently undergoing treatment and is under regular follow-up.

By revisiting the projection geometry, we felt that we have encountered a triple image artifact in regular RPR where in there were 2 lateral real images along with a central ghost image that had been superimposed on the cervical spine. The mechanism behind the image formation in RPR has been depicted in figure 5. In CBCT primary reconstruction, the effect of focal trough has been observed. There had been 2 lateral real image artifacts only when the focal trough was large (Fig. 4).

## Discussion

The working principle of RPR is by rotating a narrow beam of x-rays in horizontal plane, around an invisible rotational axis, positioned intraorally. The images captured on a RPR may be real images or ghost images. Real images can be either single or double real images. While a real image is formed when the structure is located between the rotational center of the beam and the film, the double real image occurs in the central portion of the oral and maxillofacial region in a diamond shaped zone where the objects are intercepted twice by the beam (4).

Artifacts, are often formed when an object is positioned between the x-ray source and the center of rotation. In an anatomic perspective, this object should lie behind the center of rotation. The existence of such entities were initially proposed by McDavid *et al.*, in 1983 and further experimentally demonstrated by Reuter in 1999(4). Since then many reports suggestive of this phenomenon appeared in literature (5-8). However, no reports of teeth or treatment devices producing these misleading images were reported.

In RPR, the path of the x-rays initially extends from the origin to the anterior rotation centre (ARC) (Fig. 5) and in second stage, they rotate around the ARC (Fig. 5) followed by a third stage, when the source moves from ARC to its terminal position (Fig. 5). Objects placed along the path of the rotation centre appear twice since they are continuously within the beam while structures placed in the center (Fig. 5), are portrayed once in the midline (Fig. 5) and twice symmetrically on either side of RPR (Fig. 5). Confluence of the beam in the center forms a diamond shaped area as reported by Reuter (4). This diamond shaped region corresponds to the midline of the patient from about the middle of the image to the most posterior extent of the radiograph. The double real image is a pair of real images formed by an object lying within this diamond shaped zone and they are mirror images of each other. Anatomically, structures which may produce double real images are the hard and soft palate, epiglottis and palatal torus. Besides the science behind this phenomenon, it has been reported in literature that each manufacturer have unique trough, and each individual unit may have its own characteristics. An operator needs to be familiar with the features of his machine for proper understanding of RPR imaging (6).

When abnormal structures such as a tooth or a device fall within the diamond shaped region, RPR images may guide the orthodontist towards an inaccurate diagnosis by producing a triple images. Such cases must be treated with circumspect, and apart from RPR, additional imaging modalities which includes conventional occlusal radiographs as well as 3D reconstructed CBCT images, need be employed to provide a judicious interpretation of the clinical situation.

In the present orthodontic scheme, the use of mini implants in this anatomical region is fairly common. The discerning orthodontist must be cognizant of the possible positional and analytical errors which may be prevalent in a conventional RPR.

In conclusion, with increasing use of advanced dental imaging modalities, careful interpretation is necessary. Human judgment is critical, which relays in turn on deeper understanding of the mechanism that these modalities operate on. With newer diagnostic and newer applications of existing ones, it can be expected that unknown issues may crop up and an alert clinician always can pick the abnormality if he/she is prepared for such eventualities by understanding the fundamental principles behind the technology. The field of dental imaging has a multitude of manufacturers and imaging software available to the operator. In order to provide comprehensive and accurate care to the patient, it is prudent to have sufficient knowledge about the machine, its operation and the working principles behind it.
